# Efficacy and safety of Suanzaoren decoction for primary insomnia: a systematic review of randomized controlled trials

**DOI:** 10.1186/1472-6882-13-18

**Published:** 2013-01-22

**Authors:** Cheng-long Xie, Yong Gu, Wen-Wen Wang, Lin Lu, Deng-lei Fu, Ai-ju Liu, Hui-qin Li, Ji-huang Li, Yan Lin, Wen-jie Tang, Guo-qing Zheng

**Affiliations:** 1 The Center of Neurology and Rehabilitation, The Second Affiliated Hospital of Wenzhou Medical College, Wenzhou, 325027, China; 2 School of Chinese Medicine, University of Hong Kong, Hong Kong, SAR, China; 3 Department of Psychology, School of Environmental Science and Public Health, Wenzhou Medical College, Wenzhou, 325000, China

**Keywords:** Insomnia, Suanzaoren decoction, Systematic review

## Abstract

**Background:**

Insomnia is a widespread human health problem, but there currently are the limitations of conventional therapies available. Suanzaoren decoction (SZRD) is a well known classic Chinese herbal prescription for insomnia and has been treating people’s insomnia for more than thousand years. The objective of this study was to evaluate the efficacy and safety of SZRD for insomnia.

**Methods:**

A systematic literature search was performed for 6 databases up to July of 2012 to identify randomized control trials (RCTs) involving SZRD for insomniac patients. The methodological quality of RCTs was assessed independently using the *Cochrane Handbook for Systematic Reviews of Interventions*.

**Results:**

Twelve RCTs with total of 1376 adult participants were identified. The methodological quality of all included trials are no more than 3/8 score. Majority of the RCTs concluded that SZRD was more significantly effective than benzodiazepines for treating insomnia. Despite these positive outcomes, there were many methodological shortcomings in the studies reviewed, including insufficient information about randomization generation and absence of allocation concealment, lack of blinding and no placebo control, absence of intention-to-treat analysis and lack of follow-ups, selective publishing and reporting, and small number of sample sizes. A number of clinical heterogeneity such as diagnosis, intervention, control, and outcome measures were also reviewed. Only 3 trials reported adverse events, whereas the other 9 trials did not provide the safety information.

**Conclusions:**

Despite the apparent reported positive findings, there is insufficient evidence to support efficacy of SZRD for insomnia due to the poor methodological quality and the small number of trials of the included studies. SZRD seems generally safe, but is insufficient evidence to make conclusions on the safety because fewer studies reported the adverse events. Further large sample-size and well-designed RCTs are needed.

## Background

### Description of the condition

Insomnia is a highly prevalent, often debilitating, and economically burdensome sleep disorder, associating with various situational, medical, emotional, environmental and behavioral factors [1]. It is estimated that 25% to 30% of adults have occasional sleep difficulties and about 10% encounter chronic insomnia that meet diagnostic criteria for insomnia with the presence of symptoms for at least 1 month [2,3]. Health troubles associated with insomnia include lower quality of life, greater use of medical services as well as decreased work efficiency [4]. Insomnia is also associated with higher risk of depression and augment rates of absenteeism [5,6].

Currently, a proportion of insomniac patients worldwide resort to various kinds of complementary and alternative medicine (CAM). An analysis of the United States National Health Interview Survey data from 2002 by Pearson et al. [7] revealed that 4.5% of adult population had used CAM to treat insomnia or trouble sleeping during the previous 12 months. A population-based pharmacoepidemiology survey of Chinese herbs for treating insomnia in Taiwan during 2002 indicated that the peak age of these subjects with insomnia treated by traditional Chinese medicine (TCM) was between 40 and 49 years (25.3%), followed by 30-39 years (23.8%) and 50-59 years (17.0%) [8].

### Description of the intervention

In western conventional medicine (WCM), guidelines of British association for psychopharmacology (BAP) recommend that cognitive behavioral therapy (CBT) is an effective treatment for insomnia delivered either individually or in small group format (Ia), and has been found to be as effective as prescription medications for short-term treatment of chronic insomnia (Ia) [9]. However, there is still limited evidence of clinically meaningful changes beyond reductions of insomnia symptoms such as improved daytime functioning, quality of life [10]. In addition, it remains largely underutilized because there are few trained therapists [9] and patient adherence is an issue [11]. Guidelines of BAP recommended that Z-drugs and short-acting benzodiazepines are efficacious for insomnia (Ia), and prolonged release melatonin improves sleep onset latency and quality in patients over 55 (Ib) [9]. However, benzodiazepine receptor agonists is limited by concerns regarding long-term efficacy and the potential for abuse, dependence, and adverse effects [12]. Clinical application of prolonged release melatonin was only recommended treatment of insomnia in the elderly for 3 weeks (1b) [9], and no consistent conclusion could be drawn so far [13].

TCM including Chinese herbal medicine (CHM), acupuncture and other nonmedication therapies has played an important role in the medical care of insomnia patients for more than 2000 years [14]. In a typical CHM prescription, a complex integration of two or more single Chinese herbs together formed a formula to achieve additive or synergistic effects. Based on the Chinese diagnostic patterns, i.e., inspection, listening, smelling, inquiry, and palpation, the CHM prescription follows a completely different rationale than many western drug treatments [15]. Suanzaoren Decoction (SZRD) is composed of 5 kinds of CHMs: Suanzaoren, Fuling (Poria, Hoelen), Chuanxiong (Rhizoma Chuanxiong, Ligusticum), Zhimu (Rhizoma Anemarrhenae, Anemarrhena), and Gancao (Radix Glycyrrhizae, Licorice) [16], all of which are recorded in the Chinese Pharmacopoeia (Version 2010). SZRD is one of most famous herbal prescriptions for insomnia, which firstly documented in the classical Chinese medical book JinGuiYaoLue (Synopsis of Prescriptions of the Golden Chamber) by Zhang Zhongjing (AD 152-219) at the end of the Han Dynasty [16]. In modern time, SZRD are still widely used throughout China and elsewhere in the world for the treatment of insomnia. A survey research from Taiwan [8] and a recently published systematic review [14] showed that SZRD was one of the most commonly prescribed Chinese herbal prescription for insomnia.

### How the intervention might work

In experimental studies, SZRD showed the hypnotic effect on sleep enhancement and its underlying mechanisms may be mediated through the activation of serotonergic system in additional to the activation of Gamma-aminobutyric acid A (GABA_A_) receptors in rat [17,18]. At the global metabolomics levels, SZRD can increase sleep activity and exhibit binding affinity for serotonin receptors in a model of insomnia drosophila [19]. Semen ziziphi spinosae, as the principal drug in the SZRD, has exhibited the central nervous system tranqualizing effect. Spinosin has been proposed as the active component in Semen ziziphi spinosae [20]. It has been reported jujubogenin through the hydrolysis of the saponin jujuboside A to be the effective suanzaoren constituent to interact directly with the GABA_A_ receptor [21]. In addition, the hypnotic effect of zizyphi spinosi semen may be mediated by the anticholinergic and antihistamine action of betulic acid, an active compound of zizyphi spinosi semen [22,23].

### Why it is important to do this review

Currently, prescriptions of CHMs for insomnia largely reflect the experience of the Chinese herbal doctor or what is recommended by traditional Chinese texts [8]. In the classical literature, SZRD is proposed to nourish the blood and calm the nerves to eventually bring on a tranquillizing sensation and reduce the effect of sleep disturbance [16]. Evidence-based medicine (EBM) is a strategy for the critical evaluation and uniform comparison of clinical trial data with conclusions according to predetermined efficacy criteria. Owing to the significant health risk of insomnia and the limitations of currently available conventional therapies, there have been a number of controlled studies over the past decade to evaluate the efficacy and safety of SZRD for insomnia. A systematic review addressing the efficacy of modified SZRD for insomnia has also been published recently with 13 RCTs in Chinese, and it concluded that the total effective rate in modified SZRD group was better than that in control group [24]. However, this low-quality systematic review had many shortcomings as follows and language barriers which is not readily accessible to Western scientists. For example, the authors only searched three Chinese databases from 2000 to 2010; evaluation of SZRD by comparing another form of CHMs in control group also was included; 6 of 13 studies were co-morbid insomnia as secondary to another condition; the conclusions are not scientifically sound and very misleading. Therefore, it is worthwhile to undertake a update systematic review of RCTs using SZRD as treatment for primary insomnia that was published in English.

### Objective

Given the gap between the lack of scientific evidence for the efficacy of SZRD and the widespread use and growing enthusiasm among the public possibly due to the limitations of conventional therapies available, the objective of current systematic review is an updated and English version to evaluate the efficacy and safety of SZRD for primary insomnia.

## Methods

This systematic review is conducted according to the Preferred Reporting Items for Systematic Reviews and Meta- analyses: Additional file 1 The PRISMA Statement [25].

### Eligibility criteria

#### Types of studies

Only randomized controlled trials (RCTs) which evaluate SZRD for primary insomnia were included in this review, regardless of blinding, publication status or language. Quasi-RCTs, for example, allocation by date of birth, day of the week, medical record number, month of the year, or the order in which participants are included in the study (alternation) were excluded.

#### Types of participants

Participants diagnosed with primary insomnia according to Chinese classification of mental disorders (CCMD)-2-R and the update version CCMD-3 [26] or Guideline for ClinicalTrials of New Patent Chinese Medicines (GCTNPCM) criteria [27] were included, regardless of age, gender and ethnicity. The CCMD-3 criteria is as follows: (1) one or more of the following sleep related complaints: difficulty initiating sleep, sleep not deep, waking up too early, too much dream, not easy to sleep again after waking up, uncomfortable after waking up, fatigue and daytime sleepiness; (2) the symptom of insomnia that has been present for three or more times a week and persist for at least a month; (3) marked distress due to discontent about the quantity and quality of sleep, or vocational dysfunction; (4) to exclude secondary insomnia caused by or co-morbid with physical illness and mental disorders etc. The GCTNPCM criteria is as follows: (1) Patients with the typical symptoms of insomnia: difficulty initiating sleep, often nocturnal awakenings, difficulty maintaining sleep or not easy to sleep again after waking up, wake up early in the morning, difficulty initiating sleep, the nighttime sleep difficulty, daytime sleepiness, sleep less than 5 hours; (2) a history of recurrent insomnia and persist for at least a month; (3) the degree classification of insomnia. Mild: often nocturnal awakenings or difficulty maintaining sleep, wake up early in the morning, but do not affect vocational dysfunction; Moderate: Sleep less than 4 hours, but can adhere to the work; Severe: Up all night, difficult to persist in the normal work; (4) to exclude secondary insomnia caused by or co-morbid with physical illness and mental disorders etc.; (5) adults 18-65 years of age.

#### Types of intervention

Studies which used SZRD as a monotherapy or as an adjunct therapy to conventional medicine were included. There is no restriction on dosage including frequency, dose, and intensity. The duration of treatment courses was at least 2 weeks. Comparator interventions were conventional medicine (Benzodiazepine), placebo, or no treatment. Studies comparing SZRD with another CHM were excluded. The definition of modified SZRD is at least 4 out of 5 herbs in SZRD or plus a few herbs based on differentiation of syndrome, but the prescription must include the principal drug, Semen ziziphi spinosae.

#### Types of outcome measures

The primary outcome measurement was sleep questionnaires such as the Pittsburgh Sleep Quality Index (PSQI) [28], the Spiegel sleep questionnaire [29], sleep dysfunction rating scale [30] at the end of the treatment course. The secondary outcome measurement was the clinical effective rate based on response evaluation criteria in TCM treatment of insomnia [27], and the adverse events. In Guideline for Clinical Trials of New Patent Chinese Medicines, evaluation standards for clinical therapeutic effects were as follows [27]: (1) clinical cure: sleep time to restore normal sleep time or the nighttime sleep duration of more than 6 hours, deep sleep, full of energy after waking up; (2) markedly effective: significant improvement of insomnia; sleep time increased over 3 hours compared with the previous sleep time; an increase of the depth of sleep; (3) effective: amelioration in symptoms; sleep time increased less than 3 hours compared with the previous sleep time; (4) ineffective: no significant improvement of insomnia, or deteriorated after treatment. Other assessment criteria of clinical therapeutic effect made by other authors with comparable definitions were also considered. However, we excluded the trials due to deficiency of useful data.

### Literature search

We electronically searched Cochrane Central Register of Controlled Trials (CENTRAL), PubMed, EMBASE, Chinese National Knowledge Infrastructure (CNKI), VIP information database, and Wanfang Data Information Site. The publication time is from the start of each database up to July of 2012. A manual search for conference proceedings relevant to this topic, references from relevant reports of clinical trials and review articles was performed to retrieve all potentially relevant published and unreported material. The following search strategy, using the grouped terms, was used for MEDLINE, and was modified to suit other databases.

Medline (Pubmed) search strategy.

1. exp sleep

2. sleep$.mp

3. insomnia$.mp

4. wakeful$.mp

5. sleepless$.mp

6. somnambul$.mp

7. or/1-6

8. exp Suanzaoren

9. Suanzaoren$.mp

10. Ziziphus jujub$.mp

11. Suanzaorentang$.mp

12. or/8-11

13. 7 and 12

### Study selection and data extraction

Two investigators (XCL, GY) independently reviewed the titles and abstracts to select potential references according to the criteria that established above. All full articles with potentially relevant trials were retrieved. Then, they read the selected articles independently and made a final decision for selection or not. Discrepancies were discussed and resolved by agreement or consultation with a third author (ZGQ).

A standardized data extraction form was used to extract data, including patients, methods, interventions and outcomes. The reasons for the exclusion of studies were recorded accordingly. For eligible studies, two review authors (XCL, GY) extracted the data independently. Disagreements were resolved through consultation with a third party (ZGQ).

### Risk of bias in individual studies

The methodological quality of RCTs was assessed independently using the *Cochrane Handbook for Systematic Reviews of Interventions* [updated September 2009]. Two investigators (XCL, GY) independently evaluated the methodological quality of the included articles. Disagreements were resolved through consensus or discussed with a third author (ZGQ).

## Results

### Description of studies

**Figure 1:**
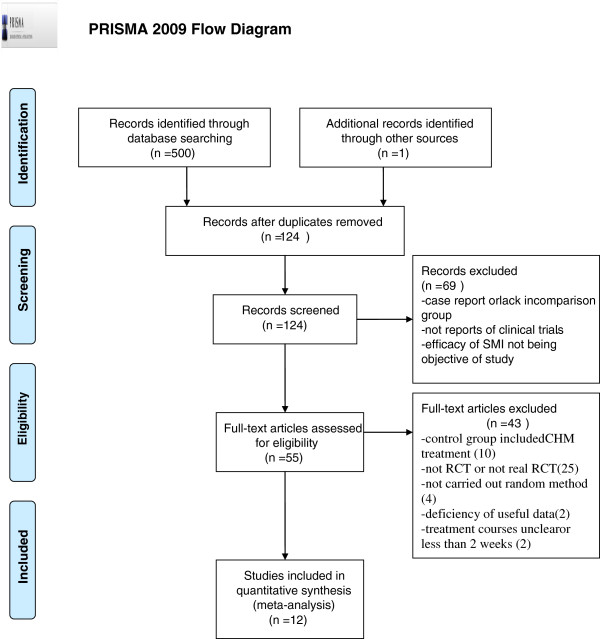
PRISMA 2009 Flow Diagram.

We identified 501 potentially relevant articles. After removal of duplicates, 124 records remained. After going through the titles and abstracts, we excluded 69 papers with at least one of following reasons: (1) not clinical trials; (2) case report or lack of control group, and (3) efficacy of SZRD not being objective of study. By reading the full text of the remaining 55 articles, 10 were excluded because control group is CHM treatment rather than WCM; 25 were excluded for not RCT or not real RCT; 4 papers were excluded as a result of not carried out random method; 2 papers were removed due to deficiency of useful data; 2 for the treatment courses being either not mentioned or less than 2 weeks. Ultimately, 12 eligible studies were selected in present study [31-42]. The screening process is summarized in a PRISMA 2009 flow diagram (Figure 1).

### Characteristics of included studies

The basic characteristics of the 12 trials are summarized in Table 1. A total of 1376 adult insomniac participants were included in these 12 trials. The disease duration ranged from 1 months [33] to 16 years [34]. 8 were diagnosed according to CCMD [32-39], 3 based on GCTNPCM criteria [31,40,41], remaining 1 were diagnosed according to both CCMD and GCTNPCM criteria [42]. All trials adopted SZRD monotherapy or adjunct therapy in the treatment group for insomnia. One study was three-group design study [42], in which both SZRD monotherapy and SZRD adjunct therapy were compared with the WCM control. Thus, 9 were monotherapy and the rest of 4 studies were combined with the WCM to treat the insomnia. All the patients in the control groups were received Benzodiazepine treatment, 4 used diazepam [33,34,37,41], 4 used estazolam [32,36,39,40], 2 used alprazolam [31,42], lorazepam [32] and oxzaepam [35] just have 1 study, respectively. The duration of treatment was varied from 14 days [31,33,39,42] to 30 days [35]. Clinical efficacy was observed in 11 studies, but the standard is different, in which 8 adopted TCM standard; 2 studies [35,42] used the reducing score rate of PSQI; 1 trial [36] applied home-made criteria. PSQI score was tested in 2 studies [37,42]. Adverse effects were reported in 3 studies [31,36,42], while no mention in the other studies (Table 1).

**Table 1:** Basic characteristics of the included studies

**Included trials**	**Eligibility criteria**	**Study designs**	**Interventions**		**Sample and characteristics (male/female; age ;Duration)**		**Outcome index**	**Intergroup differences**
			**Trial**	**control**	**Trial**	**control**		
**WU 2005**[31]	GCTNPCM in I993	RCT (method unreported) and controlled nonblind parallel study	Modified suanzaorentang 1dose/d for 2 week	alprazolam 0.4-1.2 mg/p.t qn for 2 week	70 (M:30, F:40) Mean age: Mean age: 48y Disease duration: 6 m-9y	70 (M:32, F:38) Mean age: 47y Disease duration: 6 m-9y	1. Clinical effect	1. p < 0.05
							2. adverse effect	2.P < 0.01
**Yu 2005**[32]	CCMD-III	RCT (Psychological test Numbers) and controlled nonblind parallel study	Modified Suanzaorentang 1dose/d+Lorazepam o.5 mg qn for 4 weeks	Lorazepam o.5 mg qn for 4 weeks	48 (M:22, F:26) Mean age: Mean age: 32.6±8.9y Disease duration: 1.5 m-10y	45 (M:23, F:22) Mean age: Mean age: 30.6±8.4y Disease duration: 1 m-12y	1. SDRS	1. p < 0.05
							2. HAMA	2. p < 0.05
							3. CGI	3.P > 0.05
**Tan 2006**[35]	CCMD-III	RCT (method unreported) and controlled nonblind parallel study	Suanzaorentang 1dose/d (150 ml) + Oxazepam 60 mg/d For 30d	Oxazepam 60 mg/d For 30d	N.R N.R N.R	N.R N.R N.R	Clinical effect	p < 0.05
**Yu 2006**[36]	CCMD-III	RCT (method unreported) and controlled nonblind parallel study	Baihesuan Zaorentang 1dose/d (300 ml) for 3 week	estazolam 2 mg/p.t qn For 3 weeks	63 (M:34, F:29) 63(M:34, F:29) Mean age: 31.2y Mean disease duration: 2.3y	57 (M:30, F:27) 63(M:34, F:29) Mean age: 31.3y Mean disease duration: 2.4y	1. Clinical effect	1.p < 0.05
							2.adverse effect	2.p < 0.01
**Wu 2008**[42]	CCMD-III+GCTNPCM in I993	RCT (method unreported) and controlled single-blind 3-group design study	Modified Suanzaorentang 1dose/d For 14d	alprazolam 0.2-0.6 mg/pt qn For 14d	70 (M:30, F:40) Mean age: Mean age: 48y Disease duration: 6 m-9y	70 (M:32, F:38) Mean age: Mean age: 47y Disease duration: 6 m-9y	1. Clinical effect	1. P < 0.01
							2. PSQI	2. P < 0.01
							3. adverse effect	3.P < 0.01
			Modified Suanzaorentang 1dose/d + alprazolam 0.2-0.6 mg/pt qn For 14d	alprazolam 0.2-0.6 mg/pt qn For 14d	260 (M:98, F:162) Mean age: Mean age: 48.5y Disease duration: 6 m-10y	70 (M:32, F:38) Mean age: Mean age: 47y Disease duration: 6 m-9y	1. Clinical effect	1. P < 0.01
							2. PSQI	2. P < 0.01
							3. adverse effect	3. P < 0.01
**She 2009**[37]	CCMD-III	RCT (random number table) a And controlled nonblind parallel study	Modified Suanzaorentang 1dose/d (300 ml) Divide two time for 4 week	diazepam 5 mg/pt qn For 4 weeks	60 (-,-) Mean age: 36.±8.53y Mean disease duration: 3.22±3.37y	59 (-,-) Mean age: 35.32±9.13y Mean disease duration: 3.51±3.67y	1. Clinical effect	1. P < 0.01
							2. PSQ I	2. P < 0.05
**Yuan 2009**[38]	CCMD-2-R	RCT (random number table) and controlled nonblind parallel study	Modified Suanzaorentang 1dose/d Divide two time for 15d	estazolam 2 mg/pt qn For 15d	69 (M:27, F:42) Mean age: Mean age: 37y Disease duration:	65 (M:23, F:42) Mean age: Mean age: 37y Disease duration:	1. Clinical effect	1. P < 0.05
							2. timeliness of drug	2. P < 0.05
**Luo 2009**[34]	CCMD-III	RCT (method unreported) and controlled nonblind parallel study	Suanzaorentang 1dose/d (250 ml) Divide two time For 15d	diazepam 2.5-5 mg/pt qn For 15d	30 (M:12, F:18) Mean age: Mean age: 60y Mean disease duration: 15y	30 (M:13, F:17) Mean age: 62y Mean disease duration: 16y	Clinical effect	P < 0.05
**Zou 2011**[40]	GCTNPCM in 1993	RCT (method unreported) and controlled nonblind parallel study	Suanzaorentang 1dose/d + estazolam 2 mg/pt qn For 4 weeks	estazolam 2 mg/pt qn For 4 weeks	25 (M:12, F:13) Mean age: Mean age: 48.82±4.32y Disease duration: 2 m-13 m	25 (M:13, F:12) Mean age: Mean age: 47.74±3.98y Disease duration: 1 m-13 m	Clinical effect	P < 0.05
**Cong 2011**[41]	GCTNPCM in I993	RCT (method unreported) and controlled nonblind parallel study	Modified Suanzaorentang 1dose/d Divide two time for 4 week	diazepam 2.5-5 mg/pt qn For 4 weeks	59 (M:18, F:41) Mean age: Mean age: 40y Mean disease duration: 3y	59 (M:17, F:42) Mean age: 39y Mean disease duration: 2.6y	Clinical effect	p > 0.05
**Feng 2011**[39]	CCMD-III	RCT (method unreported) and controlled nonblind parallel study	Modified Suanzaorentang 1dose/d (150 ml) qn for 2 week	estazolam 2 mg/pt qn For 2 weeks	78 (M:31, F:47) Mean age: Mean age: 39.53y Mean disease duration: 5.3mo	69 (M:25, F:44) Mean age: 38.89y Mean disease duration: 5.1mo	1. Clinical effect	1. p > 0.05
							2. Spiegel sleep questionnaire	2. P < 0.05
**Liu 2012**[33]	CCMD-III	RCT (method unreported) and controlled nonblind parallel study	Modified Suanzaorentang 1dose/d for 14d	diazepam 5 mg/pt qn For 14d	50 (M:25, F25) Mean age: Mean age: 48.5±20.3y Disease duration: 1 m-51 m	25 (M:24, F:26) Mean age: Mean age: 46.3±17.8y Disease duration: 2 m-42 m	1. Clinical effect	1. p > 0.05

*CCMD-III* Chinese classification and diagnostic criteria for mental disorders 3rd edition, *CCMD-2-R* Chinese classification and diagnostic criteria for mental disorders second edition-revision, *GCTNPCM* Guideline for Clinical Trials of New Patent Chinese Medicines, *RCT*, Randomized controlled trial, *y* year, *m* month, *d* day, *PSQI*, Pittsburgh Sleep Quality Index, *SDRS*, Sleep Dysfunction Rating Scale, *HAMA*, Hamilton Anxiety Scale, *CGI* Clinical general impression scale, *N.R* non-reported.

### Risk of bias in included studies

We utilized the criteria recommended by Cochrane Handbook for Systematic Reviews to assess the risk of bias in the 12 articles included. The number of criteria met varied from 1/8 to 3/8, all of the methodological quality are no more than 3/8 score. Although all included studies claimed randomization, only 3 articles reported the method of random sequences generation [32,37,38]. No trial described allocation concealment. Only one study reported the blinding of participants [42]. Only one trial described intention-to-treat analyses (ITT) [36]. Non-selective reporting was found in 3 trials [31,36,41]. Five studies existed certain degree other potential threats to validity. Therefore, all of the included trials were deemed to have an unclear risk of bias. The methodological quality of each study was summarized in Table 2.

**Table 2:** The methodological quality of included studies based on the Cochrane handbook

	**A**	**B**	**C**	**D**	**E**	**F**	**G**	**H**
**WU2005**[31]	?	-	-	-	-	+	+	-
**Yu 2005**[32]	+	-	-	-	-	+	?	+
**Tan 2006**[35]	?	-	-	-	-	+	-	-
**Yu 2006**[36]	?	-	-	-	-	-	+	-
**Wu 2008**[42]	?	-	+	-	-	+	?	-
**She2009**[37]	+	-	-	-	-	+	?	+
**Yuan2009**[38]	+	-	-	-	-	+	?	+
**Luo 2009**[34]	?	-	-	-	-	+	?	-
**Zou 2011**[40]	?	-	-	-	-	+	-	-
**Cong 2011**[41]	?	-	-	-	-	+	+	-
**Feng 2011**[39]	?	-	-	-	-	+	?	-
**Liu 2012**[33]	?	-	-	-	-	+	-	-

A: Adequate sequence generation; B: Concealment of allocation; C: Blinding (patient); D: Blinding(investigator); E: Blinding(assessor); F: Incomplete outcome data addressed (ITT analysis); G:Free of selective reporting; H: other potential thereat to validity. +: Yes, -: No, ?: Unclear.

## Results of individual studies

### SZRD versus placebo

No study was conducted to evaluate SZRD versus placebo control that met the included criteria. However, a self-controlled design study was 60 insomniac patients receiving placebo for one week, subsequently receiving capsules containing 1 gm of SZRD 30 min before bedtime each night for two weeks, and followed by another week of placebo administration. The results indicated that all ratings of sleep quality and well-being (sleep latency, sleep time, number of intermittent awakenings, how well slept and how felt on awakening based on seven-point scale) during active treatment were statistically significant improvements (P < 0.001) when compared with both placebo periods, and that no side effects were noted [43].

### SZRD versus western medication

There were 9 RCTs comparing SZRD monotherapy with conventional medicine (Table 1). The clinical effective rates were assessed in all 8 included studies [31,33,36-39,41,42]. Benzodiazepines was the only Western medication comparator. The clinical effective rates in the SZRD groups varied from 90% to 96.7%, with a mean of 92.5%, while the clinical effective rates in the benzodiazepine groups ranged from 66.7% to 93%, with a mean of 78.9%. However, meta-analysis could not be performed owing to the high clinical heterogeneity and low methodological quality of the included studies. Although the all 8 included studies used the clinical effective rate as an outcome measure, trials numbers are too small to draw a meaningful funnel plot [44]. Therefore, we also did not conduct the funnel plot analysis.

Based on the sleep questionnaires, one RCT reported significant effects of SZRD therapy for improving PSQI score when compared with 5 mg diazepam (p < 0.05) [37]; one RCT reported for improving score of Spiegel sleep questionnaires (P < 0.05) when compared with estazolam (p < 0.05) [39]. Interestingly, Yuan et al. [38] reported that 90 days follow up was done to gather the information of patients' sleep rebound time at the end of the 15 days of this study, and the results indicated that time to onset in SZRD group was significantly slower than in estazolam group; considerable theraputic effect was found in both groups at the seven days; the efficacy in SZRD group was better than that in estazolam group at the 14 days; in withdrawal period (16 days to 90 days), symptoms of estazolam group rebound quickly and markdly, while sleep rebound slowly in SZRD group and less that that in estazolam group. However, with the extensions of the follow up time, lost cases increased significantly.

### SZRD plus conventional medicine versus conventional medicine alone

There are 4 RCTs comparing SZRD plus Western drug therapy with Western drug therapy alone [32,35,40,42] (Table 1). According to the reducing score rate of PSQI, Wu et al. [42] indicated that there was significant difference of the reducing score rate of PSQI when compared the SZRD plus alprazolam groups with alprazolam alone groups (P<0.05). However, Tan [35] indicated that insomniac patients receiving SZRD plus Oxazepam therapy have not significantly affected the reducing score rate of PSQI when compared with Oxazepam alone (p > 0.05). Based on the clinical effective rate, Wu et al. [42] and Zou et al. [40] reported the effect of SZRD plus alprazolam or plus estazolam for improving clinical effective rate compared with alprazolam alone or estazolam alone, respectively (P < 0.05). Yu et al. [32] tested the effects of SZRD plus lorazepam compare with lorazepam alone in insomniac patients. SZRD was shown to significantly improve the score of sleep dysfunction rating scale and hamilton anxiety scale (HAMA) than that of lorazepam alone (P < 0.05). However, there was no significant difference in Clinical general impression scale (CGI).

### Adverse events

Adverse event monitoring was only reported in 3 studies [36,42], but no mention of side effects in the other 9 trials. No serious adverse effects were noted in these studies. Yu et al. [36] reported that adverse event occurred 2 cases in the SZRD group and 15 cases in the estazolam group (P < 0.01). In the study by Wu et al. [42], no adverse event was happened in the SZRD group, there were 47cases (18.1%) in the SZRD plus alprazolam group,whereas the alprazolam group occurred 34 cases (48.57%) (p < 0.01). Wu et al. [31] reported that no obvious adverse event was happened in the SZRD group, whereas there were 34 cases (48.6%) in the alprazolam group (p < 0.01).

## Discussion

### Summary of evidence

This study is the update systematic review of English and Chinese literature to determine the efficacy and safety of SZRD for insomnia. The 12 claimed RCTs with the total of 1376 insomniac patients that met the inclusion criteria. Despite the apparent reported positive findings, it is premature to conclude the efficacy of SZRD for the treatment of insomnia because the included studies were of generally poor quality and had small sample sizes. This systematic review suggests that SZRD may be safe for managing insomnia. However, only three studies reported adverse events; it is difficult to draw a definite conclusion.

### Limitations

The methodological quality of the included RCTs was generally poor. There are a number of methodological weaknesses in the primary studies. Randomization is necessary to avoid selection bias. However, only 3 articles reported the method of random sequences generation [32,37,38]. In addition, none of the included trials described allocation concealment. It has been shown that trials with inadequate concealment of allocation or unclear reporting of the technique used were on average 18% more “beneficial” than effect estimates from trials with adequate concealment (95% CI 5% to 29%) [45]. Our decision to exclude quasi-randomisation trials might need a comment. Jadad et al. [46] demonstrated that methods of allocation using date of birth, date of admission, hospital numbers, or alternation should be not regarded as appropriate. Moreover, Schulz et al. [47] found that odds reductions were exaggerated by up to 30% for trials that did not have clear concealment, and by 41% for inadequately concealed trials. We thus included the unclear randomised trial, but excluded quasi-randomised trial. We hope that “unclear” random is less risk of selection bias than that of definitely “inadequate” random.

Blinding is an essential method for preventing research outcomes from being influenced by either the placebo effect or the observer bias. However, only 1 study reported the blinding of participants [42]. Moreover, an appropriate placebo control is critical for any study exploring insomnia, mood or anxiety outcomes, as placebo-response is notoriously high in this area of study [48]. However, no placebo control was used to mask participants and care providers. Without a rigorous control for placebo effect, the results of these studies would be positive because of nonspecific placebo effects [49]. One of the main reasons for not using placebos in these trials may be the difficulties involved in preparing a liquid form (decoction) to have the same color, taste and flavor as a comparator, and thus the formulations are advised to be prepared in the form of capsule or pill [48,50,51].

ITT analysis has been considered as an important strategy in pragmatic randomised controlled trials for assuring two main purposes: (1) maintains treatment groups that are similar; and (2) it allows for non-compliance and deviations from policy by clinicians [52]. However, only 1 trial [36] stated whether they analyzed the data based on the ITT principle. In addition, information on dropout rates and withdrawal was not provided. In the absence of withdrawal reporting, information on handling of possible dropout data and the impact of dropouts on the reliability of trials is rendered suspect. Only 1 trial described the duration of follow-up clearly [38]. Lack of follow-ups led to difficulty in accounting for the long-term efficacy of SZRD treatment for insomnia.

Selective publishing and reporting are other major causes for bias, that must be considered. Non-selective reporting was found only in 3 trials [31,36,41]. It is conceivable that several negative RCTs remained unpublished and thus distorted the overall picture [53-56]. In the present study, most of the included trials reported positive results and all included trials come from China. However, some countries, including China, published unusually high proportions of positive results, and found publication bias as a possible explanation [57]. Similarly, we could not exclude the possibility of publication bias because most of the trials claimed that the tested treatments were effective, though we did not conduct the funnel plot analysis due to the small number of trials. In September 2004, the members of the International Committee of Medical Journal Editors (ICMJE) published a statement requiring that all clinical trials must be registered in order to be considered for publication [58]. However, none of included studies in this review had been formally registered. Thus, protocols were not available to confirm free of selective reporting.

The clinical heterogeneity would be very significant due to the variations in study quality, participants, intervention, control, and outcome measures. Thus, it was not possible to perform a pooling analysis of the trials.

A correct diagnosis is a crucial issue in every field lacking objective/instrumental markers of the disorder/syndrome under investigation [59]. All included studies applied national-specific diagnostic criteria (CCMD or GCTNPCM) for enrollment. CCMD-3 was the well defined diagnostic criteria for a precise clinical diagnosis of insomnia developed by the Chinese Society of Psychiatry (CSP). Moreover, GCTNPCM diagnostic criteria is not a TCM diagnosis pattern which examining the tongue, pulse, and eliciting constipation symptoms associated with TCM syndromes, but a modern criteria for insomnia of New Patent Chinese Medicines. Hence, both two diagnostic criteria specified primary insomnia and excluded comorbid insomnia. Although there is no gold standard for diagnosing insomnia, adopting some popular international diagnostic criteria such as Diagnostic and Statistical Manual of Mental Disorders (DSM-IV) and International Classification of Diseases (ICD-10) is worth promoting.

The quality control of herbal preparations is crucial for the validity of the study results. However, no trials mentioned in this important issue. To assess the efficacy of SZRD in a clinical study, all subjects should be given exactly the same intervention in terms of product identity, purity, dosage, and formulation. It is worthwhile to also point out that various benzodiazepines were used in these studies. Although belonging to the same drug class, individual benzodiazepine shows different efficacies in the treatment of insomnia [60]. Hence, a general conclusion stating the superiority of SZRD over benzodiazepines irrespective of their differences in half life, potency, dosage and adverse effect might be an inaccurate statement.

There are many outcome measures available to evaluate the effectiveness of insomnia treatments [61], and a number of outcome measures used in the studies. Unless the used outcome measures have established reliability and validity, data derived from them are subject to bias. However, the common use of “clinical efficacy rate” as an ancillary outcome measure through subjective qualitative scores such as “clinical cure”, “markedly effective”, “effective”, and “ineffective” in Chinese are not internationally recognized, and the validity and reliability of that was uncertain in assessing the outcome. Future trials to comply with international standards in the evaluation of treatment effect can solve this problem. Moreover, the time of the measurement was different among the trials, which leads to difficulties in interpreting the effects.

One of the hallmarks of a good quality trial is that it should have an adequate sample size with sufficient statistical power to detect statistical differences between treatment groups. However, there are small sample size in most of included studies. Trials with inadequate sample sizes often run the risk of overestimating intervention benefits [62]. In addition, no trials conducted pre-trial estimation of sample size, which indicated the lack of statistical power to ensure appropriate estimation of the therapeutic effect [63]. The results were likely to be underpowered [64].

No study outside of China is another weakness that potentially limited the generalizability of the findings. In addition, the use of composite outcome measures such as clinical cure, markedly effective, effective and ineffective to evaluate overall improvement of symptoms also limits the generalization of the findings.

The SZRD evaluated in this review generally appeared to be safe and well tolerated by insomniac patients. Interestingly, SZRD can possibly reduce the adverse-effect of alprazolam [42]. However, the safety of their use for SZRD could not be confirmed because only 2 studies mentioned the safety of interventions or investigated adverse events as one of the secondary outcome measures. Investigators of these studies might have underestimated possible adverse events.

### Implications for practice

Due to the poor methodological quality and high clinical heterogeneity of the included studies, and the small number of trials included in this systematic review, the current evidence is insufficient to support the efficacy of SZRD for insomnia. SZRD appeared to be well tolerated, but no confirmed conclusion can be reached because lack of RCTs assessed the safety. Therefore, current evidence from present systematic review is insufficient to recommend the routine use of SZRD for insomnia. However, It should be remembered that a lack of scientific evidence does not necessarily mean that the treatment is ineffective [65].

### Implications for research

The only extensively researched herbal medicine for insomnia to date is valerian (either as a monotherapy or in combination with kava or hops), but weak evidence for the use of herbal medicines including valerian and Kava, suggesting that further research on other herbal medicines with potential hypnotic effects is encouraged as current research in these areas is in their infancy [66]. SZRD is a well-known herb prescription for the treatment of insomnia. Although the present evidence is insufficient for support efficacy of SZRD, it is a promising candidate for further insomniac clinical trials. In addition, sufficient information about species, geographical origin of herbs, seasons of collecting and quality of the preparations should be provided [67].

Owing to a major concern in methodological quality, we recommend that the CONSORT 2010 statement [68,69], which consists of a 25-item checklist to determine study quality and rigor, should be used as a guideline when designing and reporting RCTs in the future. All clinical trials must be registered before the enrollment of the first patient, according to ICMJE statement [58]. Details of randomization and allocation concealment should be sufficiently described. The use of appropriate blinding procedures should be promoted. The development of appropriate sham or placebo controls and protocol for blinding should be considered with assessing and reporting of the success of the blinding procedures. ITT analysis is also recommended, in case of dropout and/or withdrawal. In particular, trials should have sufficiently large samples, ideally based on formal power calculations, and include appropriately long treatment periods and treatment frequencies. Widely recognized diagnostic criteria and validated primary outcome measures should be consolidated and used consistently. Attention should also be paid to the incidence of adverse events. Appropriate statistical analyses should be carried out for the baseline data and outcome results. Since insomnia may wax and wane with or without treatment, a longer followup period with serial measurements of outcomes is important to determine the genuine effectiveness and longterm effect of SZRD.

## Conclusions

There is insufficient evidence regarding the efficacy of SZRD for the treatment of insomnia because of the small number of trials and the small total sample sizes of the included trials. Another main reason to undermine the validity of findings is high clinical heterogeneity and low methodological quality of the included trials. Owing to the lack of adequate safety data, there is insufficient evidence to conclude the safety of SZRD for insomnia. Further large sample-size and rigorously designed RCTs are needed.

## Competing interests

The authors declare that they have no competing interests.

## Authors’ contributions

CLX and YG conceived and participated in its design, searched databases, extracted and assessed studies and helped to draft the manuscript. WWW, DLF and LL provided oversight of statistical methods and helped to draft the manuscript. AJL,HQL and JHL helped to search databases, extract and assess studies. YL participated in the study design and helped to draft the manuscript. WJL developed the research design and supervised all aspects of study. GQZ conceived the study, and participated in its design and helped to draft the manuscript. All authors read and approved the final manuscript.

## Pre-publication history

The pre-publication history for this paper can be accessed here:

http://www.biomedcentral.com/1472-6882/13/18/prepub

## Supplementary Material

Additional file 1 PRISMA 2009 Checklist. Click here for file
